# Selfie Dental Plaque Index: A New Tool for Dental Plaque Assessment

**DOI:** 10.4317/jced.59908

**Published:** 2022-11-01

**Authors:** Camila-Lindoni Azevedo, Paulo-Sérgio-Gomes Henriques, Claudio-Mendes Pannuti, Edgard Michel-Crosato

**Affiliations:** 1 Department of Social Dentistry, School of Dentistry, São Paulo, Brazil; 2 Professor Chairman of Periodontics, Retired, São Leopoldo Mandic, Faculty and Dental Research Center, Campinas, SP, Brazil; 3 Department of Stomatology, School of Dentistry, São Paulo, Brazil; 4 Department of Social Dentistry, School of Dentistry, University of São Paulo, São Paulo, Brazil

## Abstract

**Background:**

Plaque quantification indices are frequently used to evaluate personal oral hygiene. Education in self-care and self-diagnosis is effective in prevention and control of both dental and periodontal disease. Mobile technology has become a ubiquitous technology and can be particularly useful in the self-monitoring of health promotion. To evaluate the selfie dental plaque index compared with O´Leary index (DPI) and visible plaque index (VPI). The secondary outcome was to compare full-mouth and anterior teeth plaque index analysis.

**Material and Methods:**

A sample of 47 adults were evaluated using a four-stage protocol. All teeth (except third molars) were analyzed for VPI and DPI. A selfie Digital Camera captured the image of the patient’s smile (without and with disclosing solution), which was analyzed using Image J software (ImageJ 1.52a, National Institutes of Health). Adobe Photoshop software (Copyright © 2020 Adobe) was used for individual segmentation. The calculation of the selfie index of visible plaque (SVPI) and disclosed (SDPI) was done through the area with plaque of each image in relation to the total teeth area.

**Results:**

Spearman’s correlation test showed a moderate correlation between VPI and SVPI (rho = 0.6, *p*<0.001), whereas between DPI and SDPI the correlation was weak (rho = 0.2, p = 0.13). The correlation between the plaque index using all the teeth present, showed a strong correlation with the analysis only of the anterior teeth (rho = 0.8, *p*<0.001).

**Conclusions:**

Our results showed the potential of smiling images as a new tool for quantitative measurements and showed moderate correlation when compared with the visible plaque index. Anterior teeth provided reliable plaque indexes when compared with full mouth analysis.

** Key words:**Dental Plaque Index, dental hygiene, mHealth, health Promotion, oral Health.

## Introduction

Periodontal diseases and caries are highly prevalent worldwide. Both, cause tooth loss, are prevenTable and remains a major public health problem. Although both diseases are multifactorial, the dental biofilm is a major biological determinant common to the development of the two diseases. Thus, prevention and management of caries and periodontal diseases is based on biofilm removal through self-performed dental plaque control (1). However, adequate biofilm removal is difficult, because it requires dexterity and motivation. As a result, the majority of the patients does not present adequate levels of oral hygiene (2). Consequently, there is a need to develop and validate non-invasive diagnostic tools and preventive behavior change strategies (3).

A number of clinical plaque indices (PI) have been use for quantifying the presence of dental plaque. The Visible Plaque Index (VPI) was proposed by Ainamo and Bay (4), to assess the quality of oral hygiene through clinical observation of the presence of biofilm on dental surfaces by means of simple categorical definitions (presence or absence of plaque). In the O´leary Index (DPI) (5) teeth are stained with a disclosing solution, presence of plaque is scored on a dichotomous variable and the final score per individual is the sum of the plaque scores divided by the number of surfaces examined. Both indices assess the presence of plaque by an observer using visual examination, introducing an element of subjectivity to the process (6).

Currently, mHealth technology has been successfully implemented for different medical purposes for example, reducing sedentary behaviour (7), improving toothbrush behavior (8) and promoting oral health (9). The Quantified Self (QS) movement, which aims to improve various aspects of life and health through recording and reviewing daily activities and biometrics, is a new and upcoming practice of self monitoring that holds much promise (10). Mobile technologies have been developed to better treat chronic conditions. Designed tools based on the needs of these populations could lead to greater levels of monitoring, more active engagement in their care and personalized feedback (11).

The aim of this study was to describe a reproducible and quantitative method to measure plaque accumulation using digital smartphone image (“selfies”) through comparison with two plaque indexes (VPI and DPI) to establish the use selfies images, with and without disclosing solution, for purpose of plaque detection.

## Material and Methods

-Subjects

Forty seven adult subjects were recruited from a private dentistry clinic in the city of São Paulo, Brazil, and issued with an information sheet and consent form to sign before taking part in the trial. Inclusion criteria were participants aged 18 years or older with a previous need for periodontal treatment. Participants without anterior teeth or presence of fixed orthodontic appliances were excluded.

-Sample Characteristics

To calculate the sample size, the prevalence of gingival disease of 10% was considered (12), with a possible variation of 5%. An α = 0.01 was used, with standardized amplitude of the interval and two-tailed hypothesis test with β = 0.20. Wilcoxon test resulted in a sample of 47 participants.

-Data Collection and Analysis

Participants were evaluated using a four-stage protocol: (a) VPI, proposed by Ainamo & Bay (13); (b) smartphone digital image; (c) DPI, proposed by O’Leary (14) using fuchsin (Eviplac™); and (d) smartphone digital image with plaque disclosure. All teeth were examined on both clinical indexes (VPI and DPI) and analyzes were performed by the same examiner (CLA), during a single dental appointment. Anterior teeth were also combined into groups, resulting into two partial indexes, VPIant and DPI ant.

**Figure 1:**
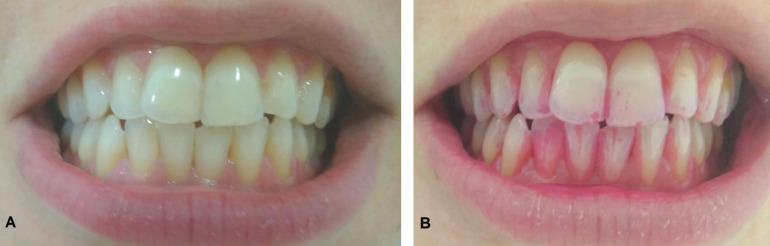
The smartphone image (A) and the smartphone plaque disclosed image (B).

The images were taken from the participant’s natural smile, using the anterior teeth that were naturally exposed in the analysis (Fig. 1A,B). The “selfie” index was performed using the image of the anterior teeth (four incisors and two canines) obtained through a smartphone camera (Xiaomi Redmi 4, 13MP). The selfie score of all teeth was estimated by applying regression analysis on the information obtained for the front teeth using the data of ten subjects under the two situations (with and without disclosing) and using a commercial camera (Canon EOS 70D, EFS 18-135mm lens, 20.9 megapixels, LED Ring).

**Figure 2:**
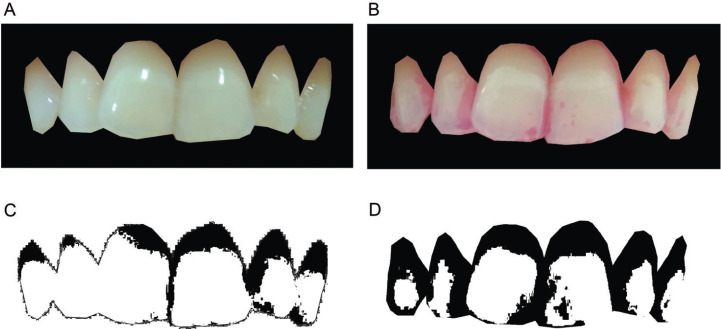
Anterior teeth image (A) and plaque disclosed anterior teeth image (B). Image processing: the plaque (in black) identified by the procedure described in this paper. Plaque area (C) and plaque disclosed area (D).

The images were saved as Joint Photographic Experts Group (JPEGs), 1600x1200 pixels in 24-bit color depth. A permanent database was created which could be used for further studies, or to re-measure for reproducibility studies if required. Images were displayed using Adobe Photoshop software (Copyright © 2020 Adobe) and the teeth area were cut with object selection tool, resulting in an image with six anterior teeth (Fig. 2A,B). A blinding single examiner (CLA) performed the cutting and selection process. First, Image J image processing software (ImageJ 1.52a, National Institutes of Health) were used to obtain the total area (TA). The color threshold was performed using the Otsu method, since: 1) it was the method of Kasai *et al*. (15), for image processing; 2) it transforms the image into binary, and 3) allow automatic image thresholding (16). Preliminary statistical analysis indicated that HSB coding was more successful in discriminating the sample classes than RGB coding. Hue and brightness were standardized (0/255) and only saturation was manipulated, until the entire area with plaque (visible or disclosure) was included in the selection (Fig. 2C,D). The plaque area score (PA) was calculated by percentage of plaque area in relation to TA.

All the data were entered and analyzed in MedCalc 19.1 (MedCalc Software bv, Ostend, Belgium; https://www.medcalc.org; 2019). Descriptive statistics are presented and discussed. A correlation test (nonparametric Spearman test) was employed to assess the difference between the above-described outcome parameters during the study intervals employing a *P* ≤ 0.05 for evaluation of significant difference.

## Results

The age of the selected patients ranged from 18 to 75 years (mean of 42 ±13), 20 were male and 28 female, with different periodontal diagnoses (healthy=16, gingivitis=23, periodontitis=9).

The ratio of the selected plaque area for the teeth area of the trimming image, was calculated by Equation (1) SVPI and (2) SDPI. The score for all teeth was estimated using multiple linear regression analysis. The correlation coefficients for the index values and between index and area are shown in Table 1.

**Table 1:**
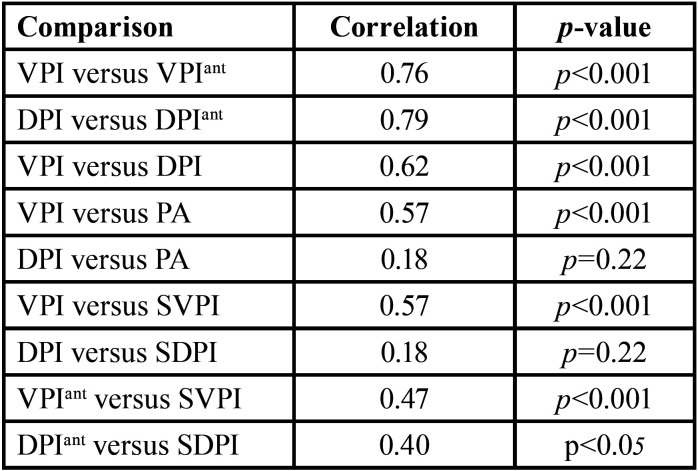
Spearman correlation between selfie index scores, area and original index scores and with original index scores.

SVPI = -7.5 + 1.14 . (PA / TA) (1)

SDPI = 40.62 + 0.44 . (PA / TA) (2)

**Figure 3:**
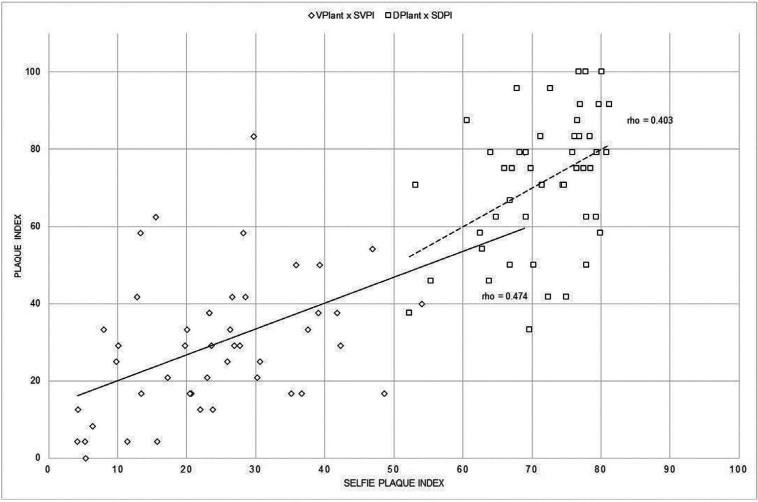
Relationship between selfies index and the plaque index without (VPIant) and with disclosing solution (DPIant).

**Figure 4:**
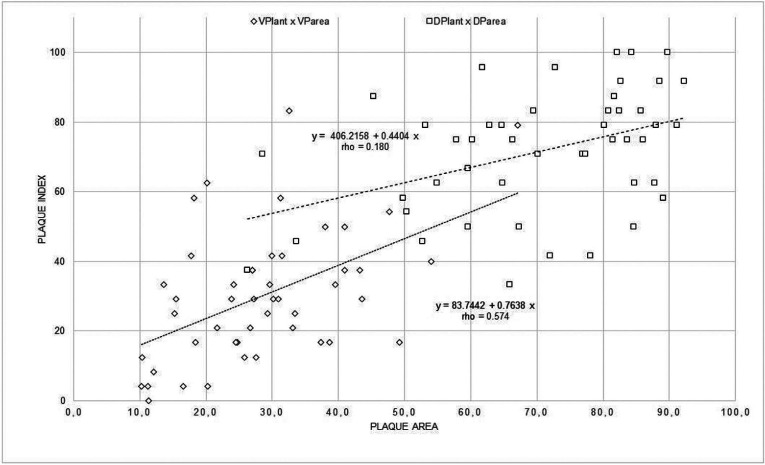
Relationship between amount of plaque of the anterior teeth using the selfie method and the visual plaque index without (VPIant) and with disclosing solution (DPIant).

The coefficient of correlation (rho) between the SVPI score and the VPI score calculated by the operator was 0.57, indicating a moderate association between them. The rho value also shows that the SDPI and the DPI calculated by the operator are not correlated. Figure 3 shows the results of a comparison between the selfies index values of the two methods and the PI value of the same teeth and method, calculated by the operator. Instead, in Fig. 4 shows the relationship between the amount of plaque related to the 6 anterior teeth as measured by the procedure described in this paper (SVPI and SDPI) and the plaque index recorded scored by VPIant and DPIant. The coefficients of determination were low (rho=0.474 and 0.403 for each index). This shows that the plaque index is not a measure of plaque quantity but of spatial distribution in parts of the teeth regardless of quantity.

## Discussion

The results of this study have shown that different numerical values are obtained using different clinical indices. The correlation between the VPI and DPI indices have been shown to be moderate (rho=0.62), overall the VPI scores being significantly lower than DPI scores (mean difference=36%). However, the correlation between VPI and VPIant and between IPC and IPCant were high and statically significant (rho=0.76 and 0.79, respectively). Anterior surfaces routinely demonstrated lower levels of dental plaque scores than the other regions of the dentition (17). Despite that, anterior teeth image analysis technique also been described with increased accuracy when comparing total plaque scores (18). Plaque assessment using the image of the anterior teeth can be extremely advantageous taking into account the simplicity of the technique, the potential for automation and self-monitoring, as well as the statistical correlation between both methods.

Mobile applications for healthcare systems are rapidly growing and evolving. They have the potential to address emerging problems on health services, including, the increasing number of chronic diseases related to lifestyle, high costs of existing national health services, the need to empower patients and families to self-care and handle their own healthcare, and the need to provide direct access to health services, regardless of time and place (19). Besides that, smartphones could be an efficient tool to disrupt with paternalism in health, offering the necessary tools and information for empowerment (20), goal-setting, self-monitoring and indeed planning can be useful in improving oral health-related behaviors (21).

Several computer-based methods have been developed and introduced into clinical trials. However, used some type of disclosing solution such as methylene blue (22) or erythrosine (15,21), in addition to high resolution camera. Considering disclosed plaque, the correlation between DPI and SDPI was very low (rho=0.22). Similar results was found by Kasai *et al*. between the DPI score and the quantity of plaque adhesion (r=0.17). However the value when each tooth was divided into four parts, was correlated with DPI (r=0.67). This may be explained by the fact that plaque index use categorical definitions, which are not strictly quantitative. For example, dividing the tooth surface into segments only represents a limited measurement because the score only accounts the segment is covered by plaque instead of amount of plaque area (22).

Besides that, dental disclosing solution are inconvenient and time consuming, especially when the patient are not cooperative. Additionally, can temporarily stain oral mucosa, the lips, and imperfections on tooth restorations´ margin broadly which are not easy to remove, representing is a major esthetic issue (23), that could contraindicate the routine use for self-monitoring. During the disclosed image processing, some selected plaque area had a minimal presence of dye that were not visually observed. As Smith *et al*. (21), we also found difficulty in defining the gingival margin on disclosed teeth. Those could represent a difficulty to fully automated techniques.

The benefits of the method presented are given by the possibility of automation, the reproducibility and the quantitative nature of the results. There was moderate correlation between VPI and SVPI (rho=0.57), but it warrants further investigation.

This study has some limitations: 1) The accuracy of plaque recognition was lower when more complex the marginal line of dental plaque and higher the percentage of dental plaque are. 2). Tooth photos obtained using different equipment may differ in color, resolution and other aspects. These differences will inevitably affect the accuracy of the acquired images and thus the accuracy of the Selfie Plaque Index. Therefore, in addition to the continued development and improvement of intelligent diagnosis ability, future research should relate the amount of dental biofilm (with least subjective criteria) to different oral health outcomes instead of a quantitative correlation. We hope that these limitations will be addressed; then, a mobile app could be used to detect not only dental plaque but also promote patient empowerment by offering health information and a self-management device.

Smartphones are already used for various activities, including for health-related purposes, by the majority of owners across all age groups. Plaque area recorded photographically provides a permanent record of plaque distribution on teeth that can be reassessed at a later date and create an image repository. Other advantages include instant feedback to patients and the possibility of archiving images for developing an Auto Machine Learning dental plaque assessment.

In conclusion, this study has shown that SVPI can estimate the VPI of all teeth from the information of the front tooth by using the plaque adhesion area and the teeth area. Our results suggest that this method can automatically estimate the VPI of all teeth by using the image of only the front teeth. There is a gap in health promotion related to dental biofilm control, with great potential for self-monitoring of plaque index through mobile technologies.
